# Late emergence of pathological oscillatory activity in the retina of the Retinitis pigmentosa model *RCS* (Royal College of Surgeons) rat

**DOI:** 10.1371/journal.pone.0324345

**Published:** 2025-05-27

**Authors:** Nruthyathi, Marie Jung, Jing Wang, Viviana Rincón Montes, Andreas Offenhäusser, Antje Willuweit, Frank Müller

**Affiliations:** 1 Institute of Biological Information Processes (IBI-1), Molecular and cellular physiology, Forschungszentrum Jülich, Jülich, Germany; 2 Institute of Biological Information Processing (IBI-3), Bioelectronics, Forschungszentrum Jülich, Jülich, Germany; 3 Institute of Neuroscience and Medicine (INM-4), Medical Imaging Physics, Forschungszentrum Jülich, Jülich, Germany; Northumbria University, UNITED KINGDOM OF GREAT BRITAIN AND NORTHERN IRELAND

## Abstract

Retinitis pigmentosa (RP) is a leading cause of blindness. The best studied models of human RP are the *rd1* and *rd10* mouse and the *RCS* rat (*Royal College of Surgeons*). In many models after degeneration of the photoreceptors, a pathological rhythmic activity of the retina as well as lowered efficiency of electrical stimulation were observed. In *rd10* retina, both events were shown to be intimately linked. Surprisingly, to our knowledge no retinal oscillations have been reported in *RCS* retina. As oscillations might interfere with the performance of therapeutic approaches to restore vision, e.g., retinal prostheses, it is important to know, whether they are a common feature of retinal degeneration. Electrical activity was recorded in retinae of 3–19 months (M3-19) old *RCS* rats *in vitro* using planar and penetrating multi-electrode-arrays. Short deflections in the local field potential resembling those observed in oscillations in *rd1* and *rd10* retinae were only sporadically found in M3 *RCS* retinae. Oscillations at appr. 2 Hz occurred more often and were more pronounced the older the animals were. Yet, even at M18-19 oscillatory periods were short and separated by long periods of non-oscillatory activity. In summary, in advanced stages of degeneration, *RCS* retinae display oscillations similar to *rd1* and *rd10* retinae. However, in *RCS* retina oscillatory periods are shorter than in mouse models and may, therefore, have escaped detection in earlier studies. These results together with results observed in non-rodent models suggest that pathological rhythmic activity is a common feature in RP models.

## Introduction

Photoreceptor degeneration is a leading cause of blindness in hereditary diseases like Retinitis pigmentosa (RP) and non-hereditary diseases like age-related macular degeneration. Both research and exploration of experimental therapies rest on animal models for RP [1]. Rodent models are of particular importance. Two important mouse models are the *rd1* and *rd10* mouse (*retinal degeneration*). In rat, two models have been studied best. One carries the P23H mutation in rhodopsin. The other important rat model is the *RCS* rat (*Royal College of Surgeons*) [2–9].

While in all these models photoreceptors degenerate, the reasons are different. In *rd1* and *rd10* mouse, a mutation in the gene encoding the ß-subunit of rod cGMP phosphodiesterase type 6 (βPDE) [2,3] affects photoreceptor metabolism, leading to cell death. Photoreceptor death results in a pronounced thinning of the outer nuclear layer (ONL, somata of photoreceptors) with differences between *rd1* and *rd10* in both degeneration onset and pace. The inner retina persists, however remodelling has been reported [1,4,5]. In both models, a pathological rhythmic activity of the retina emerges. These oscillations occur at frequencies of approx. 10–16 Hz in *rd1* and 3–6 Hz in *rd10* mice [10–12]. Oscillations are clearly visible in the local field potential (LFPs). Spiking of retinal ganglion cells (RGCs) is often phase-locked to these oscillations. In *rd10*, oscillations can wax and wane [11,13]. Similar oscillations occurring at a frequency of approx. 7 Hz were also observed in mouse models with photoreceptor degeneration induced chemically [12,14] or by UV light [15]. Hence, oscillations seem to emerge in RP models irrespective of the molecular mechanism underlying photoreceptor degeneration. In many animal models of RP, the efficacy of electrical stimulation was reduced compared to wild-type retina [10,12,13,16,17]. In *rd10* retina, both events were shown to be intimately linked. If oscillations vanished spontaneously or were abolished pharmacologically, stimulation efficiency increased to similar levels as in wild-type animals [12,13].

One therapeutical approach in retinal diseases based on photoreceptor degeneration is the use of retinal implants that stimulate the remaining cell types to elicit visual sensations in patients. Owing to the larger eyes, rat models of RP are better suited for testing retinal implants than mouse models. As the above-mentioned oscillations would significantly lower the performance of a retinal implant, it is of crucial importance to know, whether they are also found in rat models of RP. The P23H rat strains carry a mutation resulting in the substitution of histidine for proline at position 23 of the rhodopsin molecule [18]. Three strains were produced, all of which have been shown to exhibit a progressive retinal degeneration with profound loss of photoreceptors and light responses [19]. Spontaneous activity of P23H RGCs was reported to be higher at the age of 150–600 days than in age-matched wild-type rats, but decreased back to wild-type levels in older animals. However, no clear reports were found concerning pathological oscillations in this strain with the exception that rhythmic firing was reported (but not shown) for a subset of undefined P23H ganglion cells [20].

While the above-mentioned models rest on mutations in photoreceptor genes or on experimental ablation of photoreceptors, in the *RCS* rat photoreceptor loss is initiated by a mutation in the gene encoding receptor tyrosine kinase *Mertk.* One important task of the cells of the pigment epithelium is the phagocytosis of shed photoreceptor outer segments. In the *RCS* rat, this function is compromised and photoreceptors degenerate progressively [6–9,21]. Rod degeneration was reported to be nearly complete a hundred days after birth (P100) though a layer of cones might survive longer [22]. Three different *RCS* strains have been developed: two pigmented strains and one pink-eyed strain [8,22], for review see [21]. So far, pathological oscillations as they can be observed in *rd1* or *rd10* retina have not been reported in *RCS* rat [23].

In this study, we recorded *in vitro* from pigmented *RCS* rat retinae of different ages (3–19 months), using both, planar and penetrating micro-electrode-arrays (MEA). We describe that pathological oscillations reminiscent of those described in different mouse models can be recorded in *RCS* rat retina. Overall, oscillations are rarer and less steady than in mice, but occur more often and become more robust in later stages, concomitant with pronounced loss of photoreceptors.

## Materials and methods

### Animals

Inbred pigmented *RCS* (RCS-p + /LavRrrc) and wild-type rats (RCS-rdy + p + /LavRrrc) were received from Rat Resource & Research Center (RRRC, USA) and were bred in house. Wild-type animals of the strain C57BL/6J were obtained from Charles Rivers. *Rd10* mice were bred locally from breeding pairs obtained from Jackson (B6.CXB1-*Pde6b*^*rd10*^/J). In this line, the *rd10* mutation was backcrossed onto the C57BL/6J background for 5 generations before intercrossing to homozygosity. *RCS* rats as well as *rd10* mice were housed under controlled cyclic environmental conditions (12 h light/dark cycle; temperature 22°C) with food and water available *ad libitum*. All experiments were performed in accordance with the ARVO Statement for the use of animals in ophthalmic and vision research and in accordance with the German Law for the Protection of Animals and after approval was obtained by the regulatory authorities (AZ 81–02.04.2021.A111). All efforts were made to minimize the number of experimental animals and their suffering.

### Micro-electrode-arrays (MEA) recording and electrical stimulation

#### Planar MEAs.

Planar MEAs contained 60 titanium nitride electrodes (diameter 30 µm, spacing 200 µm, impedance 50 kΩ at 1 kHz) on a glass substrate (Multi Channel Systems MCS GmbH, Reutlingen, Germany). The data acquisition system (MC_Card, Multichannel system, Reutlingen, Germany) consisted of a USB MEA60-Up System, an integrated preamplifier and filter, stimulus generator STG 4002-1.6mA, and a PC. Signals were sampled at 25 kHz/channel. For electrical stimulation (single biphasic current pulses with cathodic phase first, phase duration: 400 μs, amplitude: 100 μA), several electrodes were chosen as stimulation electrodes while the other surrounding electrodes were used for recording. Before recordings, MEAs were pre-treated in a plasma cleaner (Diener Electronic GmbH + Co. KG, Germany) and coated with 0.5 mg/ml of Poly-D-lysine hydrobromide (PDL, Sigma) overnight.

#### Penetrating MEAs.

Custom-made three-dimensional (3D) penetrating *kirigami* MEAs based on parylene-C and PEDOT:PSS electrodes [24] were used for intraretinal recordings. The devices contained 16 electrodes distributed across four penetrating shanks, each 50 µm wide and 225 µm long. Each shank included one bottom electrode, along with three additional electrodes, with diameters of 25 µm (impedance of 27.5 kΩ at 1 kHz) and 15 µm (impedance of 39.1 kΩ at 1 kHz), respectively, and 10 µm vertical spacing. Additionally, two 28 µm surface electrodes (impedance of 22.2 kΩ at 1 kHz) were available between shanks. Intraretinal recordings *in vitro* were conducted using the ME2100-System (Multi Channel Systems MCS GmbH, Germany) and a 32-channel headstage (ME2100-HS32-M-3m). The NeuroNexus adapter (ADPT-NN-16/32 and ADPT-NN-32) was employed to establish a connection between the headstage and the front end of the *kirigami* probes.

### Tissue preparation

Briefly, mice (3–4 months) and rats (3–19 months) were deeply anesthetized with isoflurane and killed by decapitation under ambient room light. Eyeballs were enucleated and retinae isolated. Retinae of mice were cut into halves, retinae of rats were cut into quarters. One piece of retina was mounted with RGCs towards the electrode side of the planar MEA while the other pieces were kept in carbonate-buffered AMES solution (Sigma, pH of ~7.4), bubbled with 95% O_2 _+ 5% CO_2_. The retina on the planar MEA was continuously superfused by AMES solution. Drugs were dissolved in the AMES solution and delivered to the retina by continuous perfusion at a flow rate of 3 ml/min at room temperature. For the intraretinal recordings, the isolated piece of retina was placed ganglion cell layer facing upwards on a soft pillow within a perfusion chamber as described before [25]. Using an insertion method described for neural slices [24], intraretinal access was achieved with a penetrating MEA. The penetrating probe was initially positioned on the retinal surface, and a micromanipulator was used to guide an insertion rod towards the surface of the *kirigami* probe, pushing the penetrating shanks epiretinally.

### Pharmacology

MEA recordings were compared under physiological and pharmacological conditions in wild-type and mutant retinae. Retinae were stimulated electrically during all three phases of the experiment: 1) AMES for 20–30 min, 2) 500 µM γ-amino-butyric acid (GABA) for 10–15 min and 3) wash-out with AMES for 15–20 min.

### Data analysis

Data of planar MEAs were either used unfiltered, low-pass-filtered (50 Hz) for LFPs or high-pass-filtered (200 Hz) to analyze action potentials (AP). Unfiltered data were converted to ASCII files by the software MC-Data Tool to analyze them in MATLAB. Fast Fourier Transformation (FFT) was employed to analyze recordings using a custom-made script. The FFT signals were normalized by the maximum value to obtain similar Y-axis range for all different age groups. Stimulation efficiency was determined as spike rate ratio, by dividing the post-stimulus action potential (AP) rate (determined in a 0.4 s period following the stimulus pulse) by the pre-stimulus AP rate (determined over 8 s before stimulus pulse). Differences in stimulation efficiency were compared using the Mann-Whitney Test. Asterisks indicate the level of significance (****: p ≤ 0.0001; ***: p ≤ 0.001; *: p ≤ 0.05). All values are given as mean ± standard error of the mean (SEM).

Data of penetrating MEAs were imported and processed offline using the McsMatlabDataTools Matlab toolbox [26] and self-written scripts. HDF5 files generated by the ME2100-System were first generated. Raw traces were subjected to a bandpass filter with cut-off frequencies of 100 Hz and 3 kHz, using a sixth-order zero-phase Butterworth filter, with the objective of extracting spiking activity. A low-pass filter with a cut-off frequency of 100 Hz was applied to the data set to obtain local field potentials (LFPs).

### Histology

MEA recordings and immunohistochemistry were performed on retinae of the same animals. Retinal pieces were stained intact and flatmounted (horizontal view) as described earlier [27]. Primary antibodies were directed against long-wavelength sensitive opsin (ab5405, polyclonal, raised in rabbit, 1:800, Chemicon; RRID:AB_177456) and short-wavelength sensitive opsin (sc-14365, polyclonal, raised in goat, 1:200, Santa Cruz; RRID:AB_2236550); in addition, biotinylated peanut agglutinin (Sigma, 1:1600) was used. Secondary antibodies were: donkey anti-rabbit Cy2 (1:400; Dianova, Germany) and donkey anti-goat Alexa647 (1:200; Invitrogen, Germany). Peanut agglutinin was visualized using Streptavidin-Alexa647 (1:100; Invitrogen, Germany). Cone density was quantified in flatmounted retina pieces under high magnification in five fields of view (edge length 250 µm) along the midline of the retina and values were averaged.

## Results

**Fig 1:**
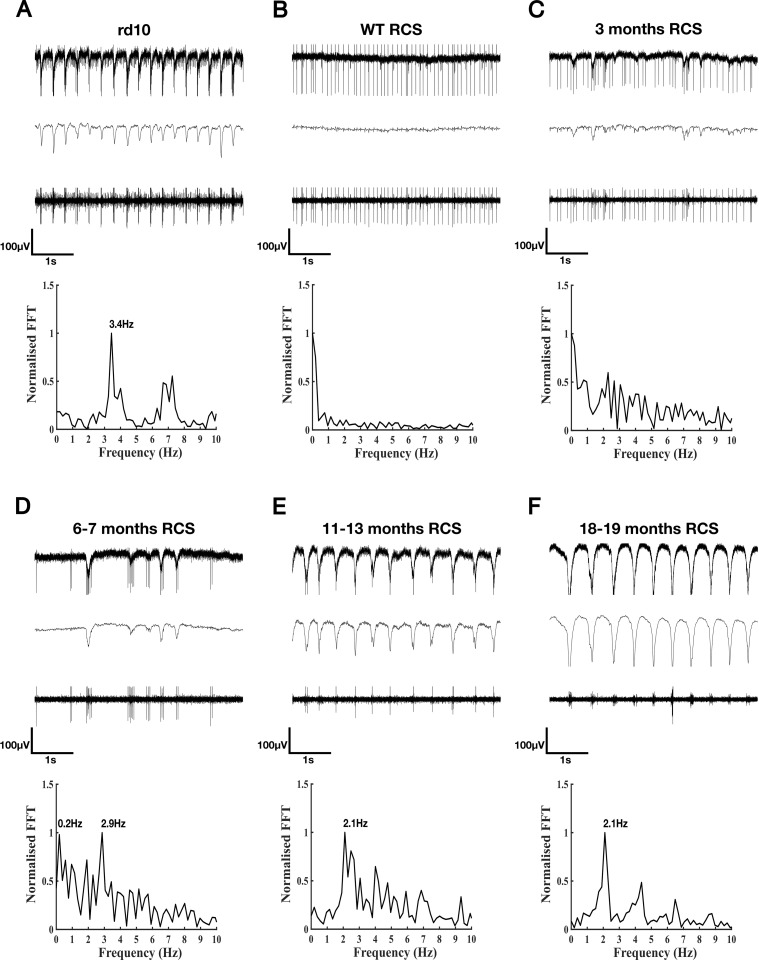
Rhythmic activity is observed in MEA recordings from *rd10* and *RCS* retina. Typical recording of neural activity from an *rd10* retina at the age of 8 months (A), wild-type (WT *RCS*) rat retina (B), *RCS* retina at the age of 3 months (C), 6-7 months (D), 11-13 months (E), and 18-19 months (F). Upper traces, unfiltered; middle traces low pass-filtered to show the LFP; lower traces, high pass-filtered to show the APs. Graphs: frequency analysis of the oscillations using FFT. Clear oscillations are observed in *rd10* retina but not in wild-type rat retina. In *RCS* retina, small and weak fluctuations are observed at 3 months. Fluctuations become more regular and more pronounced in older stages. 3 pieces from one wt *RCS*, 5 pieces from three 3 months *RCS*, 5 pieces from two 6-7 months *RCS*, 8 pieces from three 11-13 months *RCS* and 8 pieces from three 18-19 months *RCS* were evaluated.

The major research question was whether the retinae of *RCS* rats develop a pathological rhythmic activity as observed in *rd1* and *rd10* mice. Experiments were performed with *RCS* rats ranging from 3–19 months, wild-type rats and *rd10* animals. In *rd10*, oscillations were robust and reproducible. Fig 1A shows a typical recording from *rd10* retina with raw data (upper trace), low-pass-filtered to display the LFP (middle trace) and high-pass-filtered to show the action potentials (lower trace). Large negative deflections in the LFP were readily observed in *rd10* retina. Analysis using the Fast Fourier Transformation (lowest trace, FFT) revealed a frequency range for the oscillations of 3–6 Hz (3.4 Hz in Fig 1A) confirming previous results [10–12]. The frequency of oscillations could differ slightly between different retinal pieces or between different locations within the same retinal piece [17]. Typically, the FFT displayed a clear main peak as well as one or several peaks at higher frequencies, mostly appearing as second and third harmonics. Often, spike bursts were phased-locked to the negative deflections of the oscillations. In most of the retinal pieces, we observed oscillatory activity in the majority of the electrodes (60–80%). As described previously [11,13], oscillations could be present for some time in the recording, disappear, and reappear after some time. Such oscillations were never observed in wild-type mouse [11,13] or in wild-type rat retina (Fig 1B). In such cases, the FFT was basically flat.

In *RCS* rats, at 3 months (Fig 1C), for most of the time, the recordings resembled those of wild-type rats. However, occasionally, short fluctuations were observed in the LFP, that resembled fluctuations observed in *rd10*. These fluctuations were often correlated with bursting activity of RGCs. In the FFT, a broad distribution of frequencies was observed, but no peak was evident. Fluctuations were observed more often and were more pronounced the older the animals were (Fig 1D–1F). Yet, even at 18–19 months, oscillatory periods were short and were separated by long periods of non-oscillatory activity. Pronounced and regular oscillations over a time span of 2 or 5 seconds were only rarely observed. When oscillatory periods were present, peaks at around 2 Hz were observed in the FFTs (Fig 1E and 1F).

**Fig 2:**
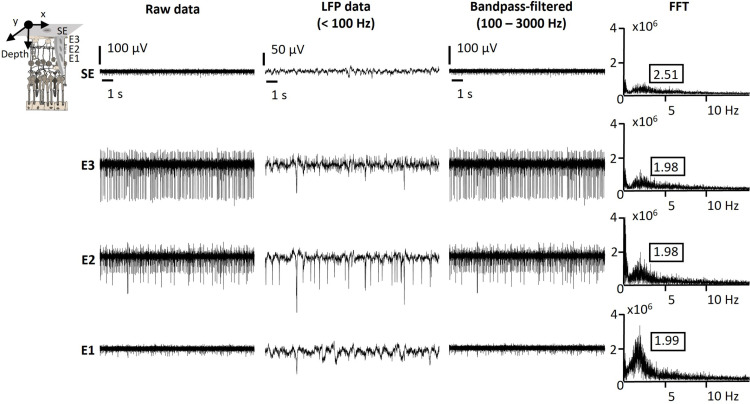
Oscillations in *RCS* rats at different intraretinal depths. Typical intraretinal recording of neural activity from an *RCS* retina at the age of 6 months recorded with a penetrating *kirigami* MEA. Raw data (first column), low-pass filtered data (second column, LFP), band-pass filtered data (third column, action potentials), and FFT analysis (fourth column) is shown for surface (SE) and penetrating electrodes (E1, E3, E3).

Furthermore, *kirigami* probes, containing both penetrating and surface electrodes were employed to capture retinal activity across retinal layers. Fig 2 shows an exemplary recording using a *kirigami* probe in a 6-month-old *RCS* rat retina, with penetrating electrodes located in the inner part of the retina. Considering that retinal ganglion cells are the only spiking neurons in the retina and considering the 80 µm sensing depth covered by the penetrating electrodes (from E1 to E3), the lower electrode (E1) likely captured low-amplitude spikes from the inner plexiform layer, while upper electrodes (E2 and E3) recorded higher-amplitude spikes from the retinal ganglion cell layer. The electrodes placed intraretinally enabled close contact with retinal ganglion cells, processes and somata of inner retinal cells, allowing us to capture pathological low-frequency oscillations around 1.98 Hz, in good agreement with the values obtained with planar electrodes (Fig 1). While the frequency was similar at different depths, the peak in the FFTs became more prominent in deeper layers. These results align with previous findings in the degenerated *rd10* retina, which demonstrated the presence of pathological low-frequency oscillations across retinal depths [17,25].

**Fig 3:**
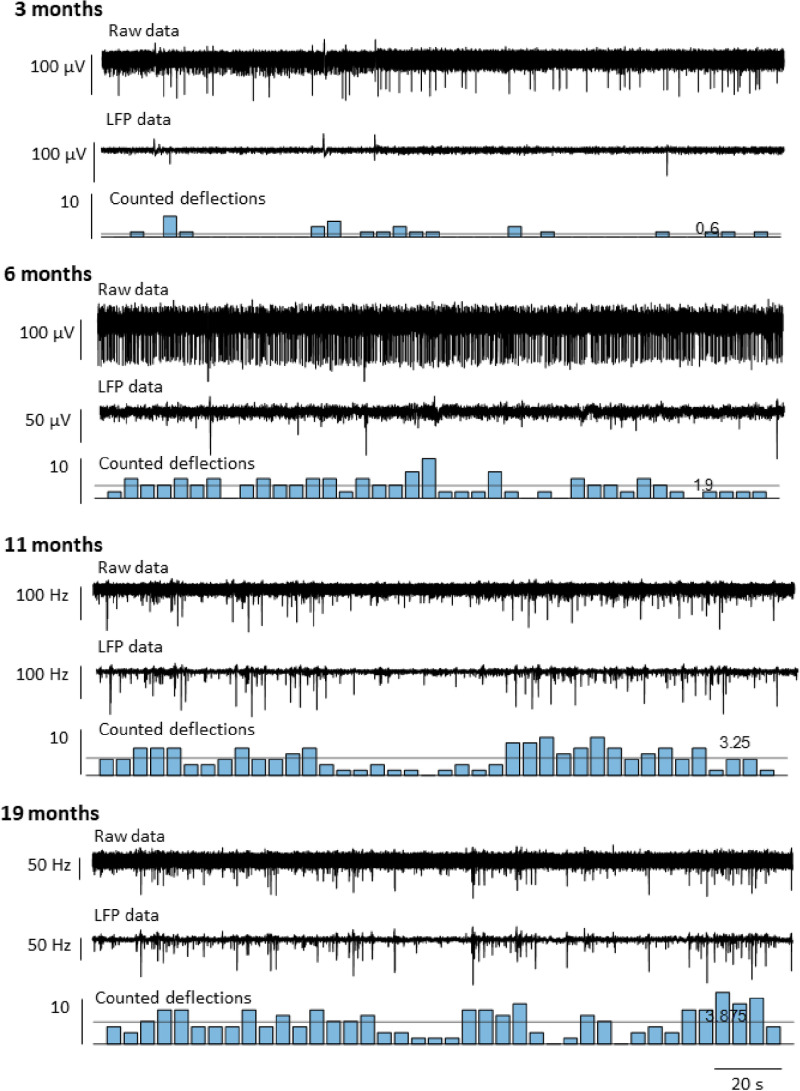
Occurrence and fluctuation of negative LFP deflections in explanted *RCS* rat retinae. Raw data, LFP data, and counted negative LFP deflections in time windows of five seconds for explanted *RCS* rat retinae at the age of 3 months, 6 months, 11 months , and 19 months.

Next, we employed penetrating electrodes located intraretinally within the ganglion cell layer to record retinal activity from explanted *RCS* retinae to get more quantitative information on the presence of oscillations at different ages. As LFP signals captured at the age of three months barely displayed pronounced oscillations, we quantified negative potential deflections within five-second time windows. As depicted in Fig 3, the frequency of LFP deflections increased with disease progression, exhibiting a fluctuating pattern with interspersed periods of absence, regardless of age. Negative potential deflections were sporadically detected, increasing from an average of 0.6 (three months) and 1.9 (6 months) to 3.25 (11 months) and 3.9 deflections (19 months) per five-second window.

In summary, in advanced stages of degeneration (11–19 months), *RCS* retinae display short periods with oscillations with a frequency of appr. 2 Hz separated by periods without oscillations. In younger stages, only individual negative deflections in the LFP are observed.

**Fig 4:**
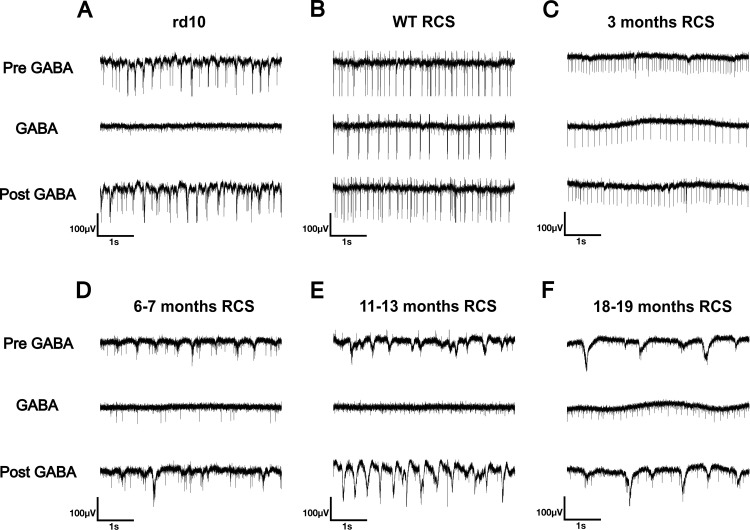
Oscillations in *rd10* and *RCS* retina are not observed in the presence of GABA. Typical recording using planar MEAs of neural activity from an *rd10* retina (A), wild-type rat retina (B), *RCS* retina at the age of 3 months (C), 6-7 months (D), 11-13 months (E), and 18-19 months (F) before application of GABA (upper trace), during GABA application (middle trace) and after washout (lower trace). GABA was applied for 10-15 min, washout for 15-20 min. Three pieces from one wt *RCS*, 5 pieces from three 3 months *RCS*, 5 pieces from two 6-7 months *RCS*, 8 pieces from three 11-13 months *RCS* and 8 pieces from three 18-19 months *RCS* were evaluated.

Next, we tested whether the oscillations behave in a similar way as those in *rd10*. In *rd10*, we could abolish oscillations by applying the neurotransmitters glycine or GABA (Fig 4A, middle trace; see also [13]). This effect was reversible upon wash-out. In a similar way, in *RCS* retina, oscillations could only be observed under control conditions, but not in the presence of GABA (Fig 4C–4F).

**Fig 5:**
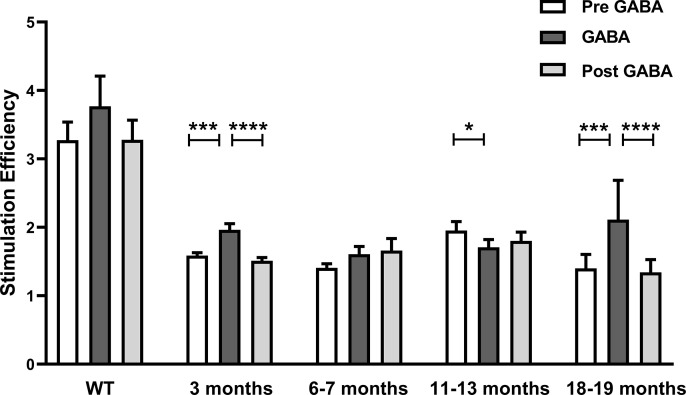
Comparison of the stimulation efficiency in wild-type rat and *RCS* retina under control conditions and during GABA application. Analyzing the stimulation efficiency, post-stimulus AP-rate was divided by the pre-stimulus AP-rate. Bar charts depict the mean ± SEM. The stimulation efficiency during pre GABA, GABA and post GABA, respectively, in wild-type (WT) was 3.27 ± 0.27, 3.77 ± 0.44, 3.28 ± 0.29, in *RCS* at 3 months was 1.59 ± 0.04, 1.96 ± 0.09, 1.51 ± 0.05, at 6-7 months was 1.4 ± 0.06, 1.6 ± 0.11, 1.66 ± 0.17, at 11-13 months was 1.95 ± 0.13, 1.7 ± 0.11, 1.8 ± 0.13, at 18-19 months was 1.4 ± 0.2, 2.11 ± 0.58, 1.34 ± 0.19. Three pieces from one wt *RCS*, 5 pieces from three 3 months *RCS*, 5 pieces from two 6-7 months *RCS*, 8 pieces from three 11-13 months *RCS* and 8 pieces from three 18-19 months *RCS* were evaluated. Mann-Whitney Rank Sum Test was used to test for significance. Differences were considered as highly significant at ****: p < 0.0001; ***: p ≤ 0.001; *: p ≤ 0.05.

In *rd10*, stimulation efficiency was low in the presence of oscillations but increased upon blockade of oscillations [13]. We tested whether the same is true for *RCS* retina. First, we observed that in *RCS* rat, independent of the age, stimulation efficiency was considerably lower than in wild-type rat (Fig 5). This correlates with the lower stimulation efficiency observed in other models of retinal degeneration [12,28–32]. In wild-type rat, stimulation efficiency was not significantly different in the presence or absence of GABA. The same was true for most recordings performed on *RCS* retina. Only at three and at 18–19 months, stimulation efficiency was slightly higher in the presence of GABA, than without. However, in contrast to *rd10* [13], stimulation efficiency in the presence of GABA did not reach wild-type level.

In *RCS* retina, oscillations became only prominent and robust at later stages of degeneration (Fig 1). One possible explanation would be that in the early stages, the number of persisting photoreceptors is sufficient to drive activity in the retina and prevent remodeling and generation of oscillations. A similar observation was reported in another retina degeneration model [33]. While for *RCS* retina it has been reported that the rods have vanished at P100 [22], little is known about the degeneration of cones. The number of sparse cones cannot be determined reliably in sections but only in flat-mounted retina. We, therefore, stained flat-mounted retinal pieces using antibodies against the two cone opsins: long wavelength sensitive (“red/green”) and short wavelength sensitive (“blue”). We also used peanut agglutinin, which was shown to label cone outer and or inner segments and endfeet in different species [27,34]. However, we found that in the later stages of *RCS* retina this marker was not reliable. We often found label of cone photoreceptor somata with opsin antibodies, but no peanut agglutinin label associated (data not shown). We, therefore, only relied on the evaluation of opsin stainings.

**Fig 6:**
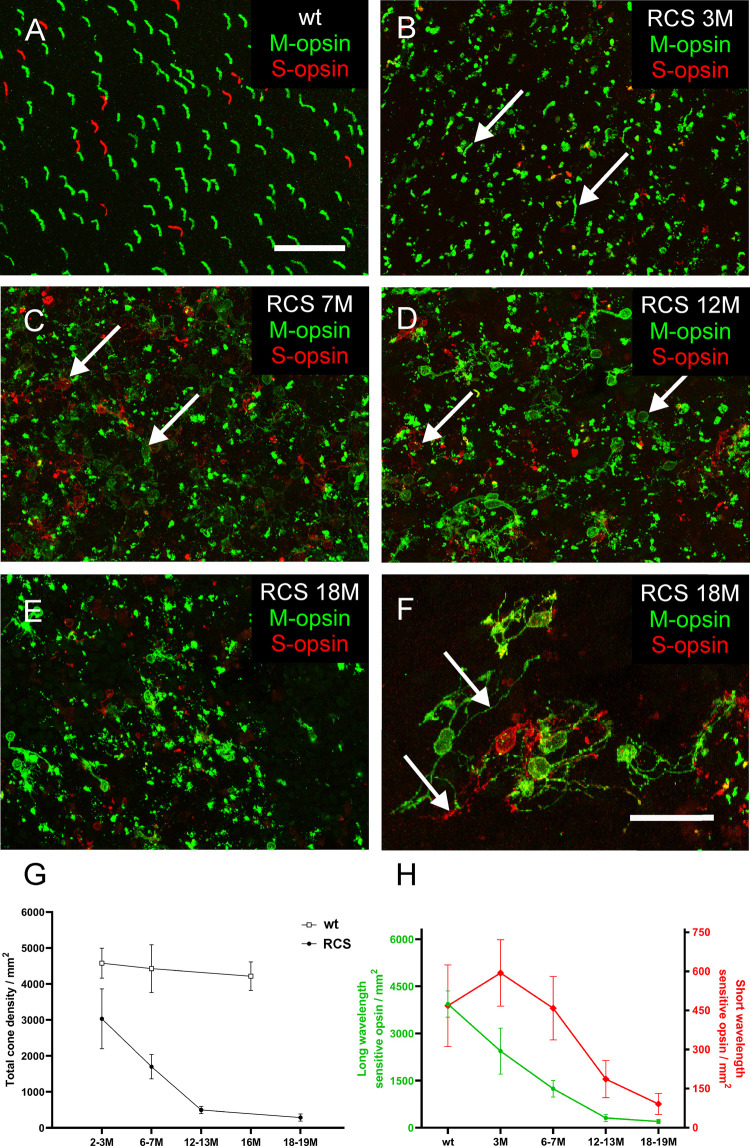
Detection of opsin expression in flat-mounted pieces of wild-type and *RCS* retina. (A) Wild-type retina displays well-formed outer segments (green: L-opsin, long-wavelength-sensitive opsin, red: S-opsin, short-wavelength-sensitive opsin). *RCS* retina at 3 months (B), 7 months (C), 12 months (D), 18 months (E, F). With increasing age, cone outer segments become more disorganized and opsin localization is shifted to the somata. Cones in old animals grow profound processes untypical of normal cones (F). Scale bars: 50 µm in A - E, 25 µm in F. (G) Total cone density/mm^2^ displayed over 19 months of degeneration in *RCS* retina. (H) Density/mm^2^ of long-wavelength-sensitive cones (green) and short-wavelength-sensitive cones (red) over 19 months of degeneration.

In wild-type rat retina, cones showed well-developed outer segments (Fig 6A). In *RCS* rat retina, during degeneration, the density of cones dropped and the morphology of cones changed (Fig 6B–6H). At three months, only some cone outer segments appeared comparable to wild-type (e.g., arrows in Fig 6B), while the majority seemed shortened or malformed. In older stages, opsin staining was found prominently throughout the somata (e.g., arrows in Fig 6C and 6D). Opsin-containing structures resembling outer segments were found. However, they did not seem to be connected to the cone somata labeled by the opsin antibodies. With progression of degeneration, many cones grew new processes (arrows in Fig 6F). The morphology of these cells barely resembled proper cone morphology. In wild-type retina, we determined over a time span of 16 months a stable total cone density of around 4500/mm^2^ (Fig 6G) with approx. 10% being short wavelength-sensitive cones (Fig 6H). In *RCS* retina, during degeneration, total cone density dropped (Fig 6G); however, the loss was mostly in the long wavelength-sensitive cones. While the number of short wavelength-sensitive cones was nearly unchanged until 6–7 months, the number of long wavelength-sensitive cones dropped to approx. 50% at three months and to approx. 25% at 6–7 months. However, even at 18–19 months, a low number of cones persisted (below 500/mm^2^; Fig 6H). Hence, cones seem to be more resilient towards degeneration than rods. However, while the somata of these cells were labeled with the anti-opsin antibodies, no outer segment could be identified.

## Discussion

For several reasons, animal models play an important role in research of retinal degeneration. First, they enable to study the underlying disease mechanisms. Second, possible therapeutic approaches can only be developed based on these models. Currently, there is no treatment for RP, but the persistent inner retina provides a target for therapies based on optogenetics, cell transplantation or electrical prostheses (for review see: [35–38]). However, success in restoring vision with prostheses depends on the functional integrity of the remaining retina. Many RP models share a common feature: the emergence of abnormal rhythmical activity or oscillations upon photoreceptor degeneration. It should be noted, that small amplitude oscillations can be recorded in normal mouse and rat retinae at different frequencies [39]. However, the pathological oscillations found in retinal degeneration models (as shown in Fig 1) are not only much larger, but often spiking activity is phase-locked to certain phases of the oscillation. This kind of oscillatory activity in the retina must be considered a major concern in retinal signal processing. First, it represents additional noise that competes with visual signals elicited by optogenetic actuators, by transplanted photoreceptors or upon electrical stimulation using a prosthesis, in particular if these signals are small [40]. Second, we recently showed that oscillatory activity does not only mask activity triggered by electrical stimulation, but actually lowers stimulation efficiency (i.e., the number of elicited spikes) in *rd10* retina considerably compared to wild-type [12,13]. Hence, oscillations might compromise the performance of retinal implants [13,40]. It is therefore important to know, whether these oscillations exist in models of RP typically used for the development of retinal implants.

Oscillations are best studied in genetic mouse models of RP. Optical recordings in the outer retina of *rd1* mice revealed oscillations around 1–2 Hz that seemed to originate from abnormal horizontal cell activity [41]. In MEA recordings of *rd1* retina, oscillations in the LFP concomitant with phase-locked spiking of RGCs at 10–16 Hz were reported. In *rd10*, oscillations looked similar but occurred at 3–6 Hz. Several lines of evidence argue that oscillations are not confined to *rd1* and *rd10* retina. First, rhythmic activity has been also reported in models in which photoreceptor degeneration had been induced pharmacologically (by injection of N-Methyl-N-Nitrosourea (MNU), oscillation frequency 7 Hz) [12] or optically (oscillation frequency 6 Hz) [15], despite a different cause and a different time course of photoreceptor degeneration. Moreover, rhythmic RGC spiking was reported in wild-type mouse retina after bleaching of photoreceptors [42]. Oscillations were also reported in bipolar cells upon pharmacological blockade of photoreceptor input [43]. Finally, abnormal rhythmic activity was found in non-rodent models upon pharmacologically induced photoreceptor degeneration in mini pig [44,45] and macaque monkey retina [46]. It is not clear whether oscillations also develop in human RP retina. However, it is worth to note that RP patients report phosphenes that could be explained by spontaneous hyperactivity [47–50]. It is, therefore, conceivable that oscillatory activity also exists in RP patients.

As oscillations seem to be a common feature in degenerated retina, it seemed surprising that no pathological rhythmic activity had been reported in rat models of RP except rhythmic firing for a subset of undefined P23H RGCs [20]. This point is of importance as rat models are better suited for the experimental insertion of retinal implants, owing to their larger eyes compared to mouse models. Here, we report oscillations in a widely used model for RP, the *RCS* rat. We found a number of similarities to retinae of *rd1* and *rd10* animals. First, oscillations were observed in the LFP. Often spiking of RGCs was phase-locked to the oscillations. Second, similar to *rd10* retina, in the presence of GABA, no oscillations were recorded. Finally, the efficiency of electrical stimulation was lower in *RCS* than in wild-type rat retina. However, there are also major differences between the oscillations in *RCS* rat and *rd* mouse. First, while oscillations in *rd10* mouse have been reported to wax and wane [11,13], once they are observed they can usually be recorded over a long time range (15–30 min) [11]. In *RCS* rat, while deflections similar to those found during oscillations could be recorded over several minutes (see, e.g., Fig 3), regular oscillations like in Fig 1 were barely observed for time spans longer than 2 seconds. Second, in *rd10*, first signs of oscillations have been reported in some studies as early as postnatal week two [51] and became more prominent 1–3 months postnatally [10–12]. Hence, oscillations occur while the retina can still respond to light (at least up to P60) [52]. In contrast, in *RCS* rat oscillations were barely observable until month 6 and became more pronounced only in late stages of photoreceptor degeneration. Interestingly, a similar behaviour was reported in another model of photoreceptor degeneration, the CNB1b knock-out [33]. In this model, rods are not light sensitive and degenerate over a long time range. Cones remain light sensitive until month 9. Oscillations only start after cones have lost their light sensitivity. The late onset of oscillations and their sporadic appearance in *RCS* rat retina may explain why they have not been reported in earlier studies.

Several models are discussed to explain the origin of the oscillations. A popular model rests on the activity of cone ON-bipolar cells and AII amacrine cells that are electrically coupled by gap junctions [43,53,54]. Choi et al. [55] reported that oscillations only occurred when the membrane potential of the AII amacrine cells was found within a range suitable to open voltage-activated sodium channels expressed in these cells. The AII amacrine cell feeds oscillatory activity in both ON and OFF channel via the ON and OFF cone bipolar cells, respectively. In contrast, another model proposed an intrinsic oscillator in amacrine cells as source for rhythmic activity [56].

We recently showed that oscillations in *rd10* retina are abolished by the inhibitory neurotransmitters GABA or glycine or by benzodiazepines acting on GABA-A receptors [13]. This effect is in agreement with both of the above-mentioned models. In the AII model, the ligands would hyperpolarize the membrane potential of the AII away from the range in which sodium channels become activated. In the amacrine cell model, inhibitory substances would reduce the rhythmic input by amacrine cells. Here, we describe that similar to *rd10* retina, no oscillations were observed in *RCS* rat retina in the presence of GABA, which may indicate a similar origin of oscillations in the two species. However, while in *rd10* retina the stimulation efficiency was increased to levels similar to wild-type retina in the presence of GABA [13], this did not occur in *RCS* retina. In *RCS* retina, stimulation efficiency was always lower than in wild-type retina with GABA having either no or only a moderate effect. In conclusion, we could show that *rd10* retina and *RCS* rat retina share the common feature of pathological oscillations. However, they differ in the time point oscillations occur for the first time, the consistency oscillations occur with, and the effect of oscillations on stimulation efficiency.
